# Supplementary material for: A set of multi-entry identification keys to African frugivorous flies (Diptera, Tephritidae)
Source: Zookeys. 2014 Jul 24;(428):97–108. doi: 10.3897/zookeys.428.7366 (PMC4143993; doi:10.3897/zookeys.428.7366)
Supplement: Supplementary material 6 — Key to Ceratitis [file zookeys-428-097-s006.zip › SF6_ZooKeys_key to Ceratitis/key/SF6_ZooKeys_key to Ceratitis/Media/Html/Ceratitis zairensis .htm]

Ceratitis (Pardalaspis) zairensis sp


***Ceratitis*** **(*Pardalaspis*)
*zairensis* De Meyer**

*Ceratitis* (*Pardalaspis*)
*zairensis* De Meyer, 1996 : 24.

Body
length: 7.0 mm; wing length: 7.0 mm.

Male

Head.
Antennal segments orange. Third antennal segment twice as long as second
segment. Arista with basal part orange coloured, otherwise dark. Frons dark
yellow, silvery over entire length but not distinct especially not in centre;
with scattered short hairs, darker than ground colour. Ocellar triangle dark.
Face yellow-orange; no median band. Occiput moderately swollen below, pale
whitish. Chaetotaxy normal for subgenus.

Thorax.
Ground colour of mesonotum pale with orange tinge; no distinct spots, except
around sockets of dorsocentral and prescutellar acrostichal setae, these
markings indistinct. Postpronotum same colour as centre of mesonotum.
Chaetotaxy normal for subgenus. Mesonotum with pale pilosity. Anepisternum with
pale pilosity except upper margin with darker hairs. Three anepisternal
bristles, median one less developed than other two. Scutellum white-yellow with
apical markings black; basally without distinct dark spots, but clearly darker
coloration.

Legs
yellow; setation typical of subgenus; predominantly pale, posterior row on fore
femur dark.

Wings
with bands mainly yellowish-brown. Banding, setation and venation normal for
subgenus. Marginal band continuous; discal band joined with marginal band;
cross-vein r-m before middle of discal cell; vein R_1_ ending beyond
cross-vein r-m.

Abdomen.
Grey-brown with orange tinge, with weakly defined spots. Pattern of spots and
setation normal for subgenus.

Female

Unknown.

(Description
after De Meyer, 1996)
